# Molecular Survey on the Occurrence of Tick-Borne Bacteria in Wild Birds from Central Italy

**DOI:** 10.3390/vetsci11070284

**Published:** 2024-06-24

**Authors:** Fabrizio Bertelloni, Giulia Cagnoli, Paolo Interrante, Renato Ceccherelli, Valentina Virginia Ebani

**Affiliations:** 1 Department of Veterinary Sciences, University of Pisa, 56124 Pisa, Italy; fabrizio.bertelloni@unipi.it (F.B.); giulia.cagnoli@vet.unipi.it (G.C.); p.interrante@virgilio.it (P.I.); 2 CRUMA-LIPU, 57121 Livorno, Italy; apusvet.cruma@libero.it; 3 Centre for Climate Change Impact, University of Pisa, 56124 Pisa, Italy

**Keywords:** *Anaplasma phagocytophilum*, *Bartonella*, *Borrelia burgdorferi*, *Chlamydia psittaci*, *Coxiella burnetii*, *Ehrlichia canis*, *Francisella tularensis*, *Rickettsia*

## Abstract

**Simple Summary:**

Birds are known to be carriers of ticks, both Argasidae and Ixodidae, which often harbor bacterial pathogens. Climatic changes observed in the last years have influenced tick distributions in several geographic areas and the migratory behaviors of many avian species; consequently, wild birds can be responsible for the introduction of ticks and relative pathogens, most of which are zoonotic, in new environments. Some studies have been carried out to detect tick-borne bacteria in ticks removed from birds worldwide, but surveys on the presence of these pathogens directly in avifauna are very scanty. This study evaluated the occurrence of tick-borne bacteria, such as *Anaplasma phagocytophilum*, *Bartonella* spp., *Borrelia burgdorferi* sensu lato, *Chlamydia psittaci*, *Coxiella burnetii*, *Ehrlichia canis*, *Francisella tularensis*, and *Rickettsia* spp., in avian spleen samples, and the obtained results suggested that wild avifauna may be involved in the epidemiology of some of the investigated pathogens.

**Abstract:**

Birds are known to be carriers of ticks infected by tick-borne pathogens, including bacteria. However, not many studies have been carried out on avian tissues to detect these agents. The aim of the present survey was to investigate, using PCR, the presence of *Anaplasma phagocytophilum*, *Bartonella* spp., *Borrelia burgdorferi* sensu lato, *Chlamydia psittaci*, *Coxiella burnetii*, *Ehrlichia canis*, *Francisella tularensis*, and *Rickettsia* spp. in the spleens collected from 300 wild birds of different orders and species from Central Italy. A total of 53 (17.67%) samples were PCR positive for at least one investigated pathogen. One (0.33%) bird was positive for *Bartonella* spp., five (1.67%) birds were positive for *C. burnetii*, eleven (3.67%) for *B. burgdorferi* s.l., and thirty-six (12%) for *C. psittaci.* No coinfection was detected. All samples were negative for *A. phagocytophilum*, *E. canis*, *F. tularensis*, and *Rickettsia* spp. The findings showed that wild birds may harbor different zoonotic tick-borne bacteria; therefore, they can contribute to the diffusion of these agents.

## 1. Introduction

Birds are known to be carriers of ticks: both Argasidae and Ixodidae; however, the most common tick species associated with avian hosts in various European areas is *Ixodes ricinus* [1]. *Borrelia burgdorferi* sensu lato (s.l.), *Anaplasma phagocytophilum*, and different *Rickettsia* species are bacterial tick-borne pathogens, which are responsible for diseases in animals and humans and are most frequently found in *I. ricinus*. Other tick-borne pathogens can be transmitted by *I. ricinus*, but also by other tick species, including *Rhipicephalus sanguineus*, *Amblyomma* spp., *Hyalomma* spp., and *Haemaphysalis* spp., which have been found on wild birds [2,3,4]. Among these agents, members of the genus *Bartonella*, *Coxiella burnetii*, *Ehrlichia canis*, and *Francisella tularensis* are bacteria of interest for human and veterinary medicine [5].

Climatic changes observed in the last years have influenced tick distributions in several geographic areas but have also influenced the migratory behaviors of many avian species [6]; consequently, wild birds can be responsible for the introduction of ticks and relative pathogens in new environments. 

Some studies have been carried out to detect tick-borne bacteria (TBB) in ticks removed from birds worldwide [7], but surveys on the presence of these pathogens directly in avifauna are very scanty [8]; therefore, the role of birds as reservoirs of TBB has not been fully elucidated.

To the best of our knowledge, data regarding TBB in avifauna from Italy regard the detection of these pathogens in ticks removed from birds [1,9,10,11], whereas information about the occurrence of the same agents in avian blood or organs are rare. A previous study regarded feral pigeons from Central Italy and found that 23.8% of the analyzed animals were infected by TBB, in particular *Bartonella* spp., *C. burnetii*, *Rickettsia* spp., *B. burgdorferi* s.l., and *Chlamydia psittaci* [12].

Considering the paucity of data on TBB infections in Italian avifauna, the aim of the present investigation was to evaluate the occurrence of some tick-borne bacteria, most of which are zoonotic, in wild birds belonging to different orders and species from Tuscany, Central Italy. In particular, molecular analyses were carried out on avian spleen samples to detect *A. phagocytophilum*, *Bartonella* spp., *B. burgdorferi* s.l., *C. burnetii*, *Ehrlichia canis*, *Francisella tularensis*, and *Rickettsia* spp. The same samples were also tested to detect *C. psittaci* DNA in view of its zoonotic importance and because chlamydia can be transmitted by hematophagous arthropods as well [13,14].

## 2. Materials and Methods

### 2.1. Samples

From 2016 to 2020, a total of 300 spleen samples were collected from dead wild birds of different species and orders. One hundred and three birds belonging to 11 avian species were found dead from trauma or predation and collected at a wildlife recovery center located in Tuscany (Central Italy); they were then transported to the Department of Veterinary Sciences of Pisa University (Pisa, Italy) for educational activities during which spleens were sampled. Only carcasses in good condition of birds dead for approximately 24–48 h were included in the survey. No ectoparasites were detected on these carcasses. One hundred and fifty-seven birds, belonging to 12 waterfowl species and 40 pheasants (*Phasianus colchicus*), were hunted during regular hunting seasons in Tuscany, and their spleens, collected by hunters, were sent, at 4 °C, to the same department. All spleens were stored at −20 °C until molecular analyses were performed. A part of the waterfowl samples (133/157) was tested for *C. burnetii* and *F. tularensis* in a previous survey [15].

No animals were specifically sacrificed for the study; therefore, no appropriate approval was necessary. 

### 2.2. Molecular Analyses

For each sample, the DNA was extracted from approx. 10 mg of spleen with the DNeasy Tissue kit (Qiagen GmbH, Hilden, Germany) according to the manufacturer’s instructions; extraction controls to monitor crosscontamination of samples were included. DNA samples were stored at 4 °C until used as templates for the PCR assays.

Different PCR protocols were employed to detect *A. phagocytophilum*, *Bartonella* spp., *B. burgdorferi* s.l., *C. psittaci*, *C. burnetii*, *E. canis*, *F. tularensis*, and *Rickettsia* spp. Negative and positive controls were added in each PCR assay. Sterile distilled water was used instead of DNA in the negative control. DNA samples extracted from slides used for indirect immunofluorescent assay (Fuller Laboratories, Fullerton, CA, USA) were used as positive controls.

All PCR amplifications were performed using the EconoTaq PLUS 2× Master Mix (Lucigen Corporation, Middleton, WI, USA) and a SimpliAmp™ Thermal Cycler (Applied Biosystems, Waltham, MA, USA). Protocols and primers previously reported were used for the detection of each pathogen and summarized in Table 1.

PCR products were analyzed by electrophoresis on 1.5% agarose gel at 100 V for 45 min; gels were stained with ethidium bromide and observed. SharpMass™ 100 Plus Ladder (Euroclone, Milano, Italy) was added as DNA marker.

## 3. Results

A total of 53 (17.67%) samples were PCR positive for at least one investigated pathogen. In detail, all samples were negative for *A. phagocytophilum*, *E. canis*, *F. tularensis*, and *Rickettsia* spp. One (0.33%) *Pica pica* sample was positive for *Bartonella* spp.; five (1.67%) samples were positive for *C. burnetii*, eleven (3.67%) for *B. burgdorferi* s.l., and thirty-six (12%) for *C. psittaci*. No coinfections were found. Detailed results in relation to the avian species are reported in Table 2.

## 4. Discussion

The findings of the present survey suggest that wild birds may harbor TBB, although low prevalences were detected and only for some pathogens. These results could be influenced by the quality of the analyzed samples—mainly the spleens from the birds found dead; in fact, even though only carcasses in good condition of the dead birds were involved in the study, their tissues could have been altered in the period between the animals’ deaths and the sampling period. In addition, the results could have been influenced by the PCR protocols used that, mainly for end-point PCRs, could not be sufficiently sensitive.

The detection of one Eurasian magpie that was positive for *Bartonella* confirms that avian species may harbor bacteria of this genus. Few studies reported bartonellae in birds, and the exact role of avifauna in the epidemiology of these bacteria has not been fully elucidated; as well, the potential pathogenicity of bartonellae for birds is not known. However, some investigations detected bartonellae in avian ectoparasites [26] and/or in birds. In North Carolina (USA), *B. henselae* was amplified from two northern mockingbirds (*Mimus polyglottos*) and one red-winged blackbird (*Agelaius phoeniceus*), as well as *B. koehlerae* from a red-bellied woodpecker (*Melanerpes carolinus*) and a common loon (*Gavia immer*) [27]; in Canada, *B. vinsonii* subsp. *berkhoffii* was found in 2% of 42 Ross’s geese (*Anser rossii*) [28]. Prevalences of *Bartonella* spp. in three bird species were evaluated in the USA: a 33% (2/6) rate was found in eastern bluebirds (*Sialia sialis*), 39% (19/49) in purple martins (*Progne subis*), and 83% (5/6) in tree swallows (*Tachycineta bicolor*) [26]. Recently, bartonellae bacteria were detected in the blood of tropical wild birds in Brazil; in particular, 19/500 (3.8%) avian blood samples were PCR positive for *Bartonella* spp. related to *B. machadoae* and *B. henselae* [29]. 

The positive results for *C. burnetii* found in this survey corroborate the results of previous studies and highlight the role of wild avifauna in the epidemiology of this pathogen. *Coxiella burnetii* was found in hematophagous arthropods sampled from birds [30,31], as well as from the droppings and tissues of different wild avian species, mainly pigeons, Passeriformes, and waterfowl [32]. *Coxiella burnetii* was detected in one seagull and some waterfowl, which could have contracted the pathogen from tick bites, although it is more likely to have been contracted through the oral route. 

Our positive results for *C. psittaci* are not surprising, but they confirm the spreading of the pathogen among wild birds of different orders and species. The transmission of this agent can occur through hematophagous arthropods [13,14], but more frequently through the inhalation and/or ingestion of contaminated material [33]. Its detection in pigeons highlights the importance of these birds as a source of infection in urban areas, where Columbiformes are largely present. Moreover, the presence of *C. psittaci*, as well as of *C. burnetii*, in waterfowl is relevant, because the carcasses of hunted animals are usually manipulated by hunters, also in domestic environments, without precautions to avoid possible infections. In addition, the detection of *C. burnetii* and *C. psittaci* is particularly relevant, because both pathogens can be dispersed through the feces of infected birds contributing to environmental contamination.

The positive results for *B. burgdorferi* s.l. confirm that the pathogen is able to infect birds. *Borrelia burgdorferi* s.l. is transmitted by ticks mainly of the *Ixodes* genus, and it causes Lyme disease, which is characterized by severe clinical forms in humans; the pathogen is a relevant concern in veterinary medicine too, because it affects horses and dogs [18]. Avifauna are known to maintain and spread spirochaetes of the genus *Borrelia*, mainly carrying ticks infected by these bacteria [34]. In some cases, borreliae were detected in avian tissue samples, and, on the basis of these findings, it has been supposed that the bird host competency for maintaining and transmitting borreliae may vary in different bird species [35,36]. In the present study, *B. burgdorferi* DNA was detected in *P. pica*, *C. cornix*, and *P. colchicus*, which are species that live in wooded areas where tick populations are abundant, and, therefore, birds are more easily exposed to the risk of infection. Unfortunately, the concentration of the *B. burgdorferi* amplicons, as well as of the *Bartonella* amplicon, obtained in our study was not enough for sequencing analyses; therefore, it was not possible to identify the species.

Surveys carried out in Europe found *A. phagocytophilum*-positive *I. ricinus* collected from migratory birds, although at a low prevalence [37]. Our finding suggest that wild avifauna do not act as reservoir of *A. phagocytophilum*, as has also been corroborated by the results of previous investigations on avian blood samples. In fact, Skotarczak et al. [38] detected no positive blood samples among the 84 that were analyzed in Poland, and Hornok et al. [8] found only the blood sample from one *Turdus iliacus*, among 128 wild birds examined, to be PCR positive for *A. phagocytophilum* in Hungary. Conversely, Keesing et al. [39] suspected the reservoir competence of some avian birds, which were analyzed in USA between 2008 and 2010 and calculated as the average percentage of ticks infected per individual host. On the basis of this calculation, they found that 33% (6/18) of American robins (*Turdus migratorius*), 43% (9/21) of veeries (*Catharus fuscescens*), 50% (7/14) of gray catbirds (*Dumetella carolinensis*), and 50% of (14/28) wood thrushes (*Hylocichla mustelina*) tested positive for *A. phagocytophilum* [39]. Similarly, De la Fuente and collaborators [40] found *A. phagocytophilum* DNA in the blood of 10/46 (22%) birds of different species.

*Ehrlichia canis* is a well-known canine tick-borne pathogen transmitted by *R. sanguineus*. It has been investigated in different canids, and the susceptibility of cats has been proven [41]. The disease in humans seems rare but possible [42]. Conversely, no information about the relation between *E. canis* and birds is available. Machado et al. [43] investigated Anaplasmataceae migratory and carnivorous birds in Brazil; *Ehrlichia* DNA closely related to an *Ehrlichia* species found in wild felines [44] was detected in an Orinoco goose (*Neochen jubata*), and an *Ehrlichia* strain closely related to *E. canis* was found in a vulture (*Coragyps atratus*). More recently, Hornok et al. [45] identified bacteria closely related to *Neorickettsia helminthoeca* and *Ehrlichia chaffeensis* in a Eurasian teal (*Anas crecca*) and a song thrush (*Turdus philomelos*), respectively. 

No samples were positive for *Rickettsia* spp. Birds have been demonstrated as possible reservoirs of rickettsiae in studies carried out in different geographic areas. *Rickettsia helvetica* DNA was found in the blood of robins (*Erithacus rubecula*) and dunnock (*Prunella modularis*) in Hungary [8]. In addition, *Rickettsia* spp. DNA was detected in wild birds of different species in Europe and the USA [12,46,47]. Conversely, more numerous studies are about the presence of *Rickettsia* DNA in ticks removed from birds [48]. 

The negative results for *F. tularensis* are in accordance with the very low prevalence of this pathogen in Italy [49]. However, considering that migratory birds come from far territories where *Francisella* can be more widely spread, the monitoring, when possible, of this agent responsible for a severe zoonosis (tularemia) is pivotal. Raptors and hooded crows were found to be resistant to *F. tularensis* in experimental infection [50], and it is generally supposed that avian species are less sensitive to this agent than mammals, probably due to their higher body temperature [51]. However, the role of birds in the dissemination of the pathogen is still under study, which is also in view of the report of tularemia in a hunter scratched by a buzzard (*Buteo buteo*) [51].

## 5. Conclusions

Birds can harbor different pathogens, including tick-borne bacteria. However, most of the data available in literature prove that avian species are carriers of TBB-infected ticks, but only a few investigations have been focused to verify TBB infection in birds, and most of them mainly regard *B. burgdorferi*. Therefore, further studies are necessary to clarify the role of birds as reservoirs of different bacterial species transmitted by ticks, which must also consider that the finding of TBB in ticks could be simply related to the presence of the pathogens in the gut blood reaming from the last feeding on a host [47]. Further studies to better verify the relation between wild birds and some pathogens, such as *A. phagocytophilum*, *Bartonella* spp., *Rickettsia* spp., and *F. tularensis*, are necessary from a One Health perspective. The detection in this study of birds that tested positive for *C. burnetii* and *C. psittaci* confirms the importance of avian population in the spreading of these zoonotic pathogens that usually are excreted in droppings contaminating rural, periurban, and urban areas; moreover, this finding highlights that several avian species, including game and synanthropic birds, may be sources of infection for humans and other animals. 

Testing available avian blood or tissues for TBB, of veterinary and human interest, contributes to improve the knowledge on epidemiological scenarios, which are constantly changing in relation to climatic changes and animal populations’ movements. 

## Figures and Tables

**Table 1:** Target genes, primers, and annealing temperature for the PCR assays carried out to detect DNA of each pathogen.

Pathogen	Target Gene	Primers Name	Primers Sequences (5′–3′)	Amplicons (bp)	Annealing Temperature (°C)	Ref.
*Anaplasma phagocytophilum*	*16 S rRNA* *	GE3a GE10	CACATGCAAGTCGAACGGATTATTC TTCCGTTAAGAAGGATCTAATCTCC	932	55	[16]
	*16 S rRNA* **	GE9f GE2	AACGGATTATTCTTTATAGCTTGCT GGCAGTATTAAAAGCAGCTCCAGG	546	55	
*Bartonella* spp.	*16S rRNA*	P12B P24E	GAGATGGCTTTTGGAGATTA CCTCCTTCAGTTAGGCTGG	296	55	[17]
*Borrelia* *burgdorferi* s.l.	*23S rRNA*	JS1 JS2	AGAAGTGCTGGAGTCGA TAGTGCTCTACCTCTATTAA	261	39	[18]
*Ehrlichia canis*	*16S rRNA* *	ECB ECC	CGTATTACCGCGGCTGCTGGCA AGAACGAACGCTGGCGGCAAGCC	478	55	[19]
	*16S rRNA* **	HE3 ECA	TATAGGTACCGTCATTATCTTCCCTAT CAATTATTTATAGCCTCTGGCTATAGGAA	389	55	[20]
*Chlamydia* *psittaci*	*ompA* *	191CHOMP CHOMP371	GCIYTITGGGARTGYGGITGYGCIAC TTAGAAICKGAATTGIGCRTTIAYGTGIGCIGC	576–597	50	[21]
	*ompA* **	218PSITT CHOMP336s	GTAATTTCIAGCCCAGCACAATTYGTG CCRCAAGMTTTTCTRGAYTTCAWYTTGTTRAT	389–404	60	
*Coxiella burnetii*	*IS1111*	Trans-1 Trans-2	TATGTATCCACCGTAGCCAGT CCCAACAACACCTCCTTATTC	687	64	[22]
*Francisella* *tularensis*	*TUL4*	TUL4-435 TUL4-863	TCGAAGACGATCAGATACCGTCG TGCCTTAAACTTCCTTGCGAT	400	60.5	[23]
*Rickettsia* spp.	*ompA*	Rr 190.70p 190-701	ATGGCGAATATTTCTCCAAAA GTTCCGTTAATGGCAGCATCT	632	46	[24]
	*gltA*	RpCS.877p RpCS.1258n	GGGGGCCTGCTCACGGCGG ATTGCAAAAAGTACAGTGAACA	381	48	[25]

Legend. *: first step of a nested PCR protocol; **: second step of a nested PCR protocol.

**Table 2:** PCR results for *Bartonella* spp., *Borrelia burgdorferi* s.l., *Coxiella burnetii*, and *Chlamydia psittaci* in relation to the investigated avian species.

					No. Positive (%)		
Family	Common Name	Scientific Name	No. Examined Spleen	*Bartonella* spp.	*Borrelia* *burgdorferi*	*Coxiella* *burnetii*	*Chlamydia psittaci*
Corvidae	Eurasian magpie	*Pica pica*	45	1 (2.22)	6 (13.33)		
	hooded crow	*Corvus cornix*	25		3 (12.00)		2 (8.00)
Ardeidae	heron	*Ardea cinerea*	2				1 (50.00)
Scolopacidae	snipe	*Gallinago gallinago*	6				1 (16.67)
Strigidae	owl	*Athene noctua*	1				
Accipitridae	Eurasian sparrowhawk	*Accipiter nisus*	4				1 (25.00)
Falconidae	falcon	*Falco peregrinus*	1				
	kestrel	*Falco tinnunculus*	3				1 (33.33)
Phasianidae	pheasant	*Phasianus* *colchicus*	40		2 (5.00)		
Columbidae	wood pigeon	*Columba palumbus*	3				2 (66.67)
	pigeon	*Columba livia*	3				3 (100.00)
Laridae	gull	*Larus marinus*	10			1 (10.00)	
Anatidae	Eurasian teals	*Anas crecca*	80			3 * (3.75)	15 (18.75)
	mallard	*Anas* *platyrhynchos*	27				2 (7.40)
	garganey	*Anas querquedula*	1				
	pintail	*Anas acuta*	4				
	greylag goose	*Anser anser*	1				
	gadwall	*Mareca strepera*	2				
	Eurasian wigeon	*Mareca penelope*	24			1 * (4.17)	4 (16.67)
	common shelduck	*Tadorna tadorna*	4				1 (25.00)
	shoveler	*Spatula clypeata*	10				2 (20.00)
	common pochard	*Aythya ferina*	1				1 (100.00)
	tufted duck	*Aythya fuligula*	1				
Rallidae	Eurasian coot	*Fulica atra*	2				
Total			300	1 (0.33)	11 (3.67)	5 (1.67)	36 (12.00)

Legend. *: samples resulted positive in a previous survey [15].

## Data Availability

The data presented in this study are available within the article.
